# State of perinatal mental health care in the WHO region of Europe: a scoping review

**DOI:** 10.3389/fpsyt.2024.1350036

**Published:** 2024-03-13

**Authors:** Anna Horakova, Hana Nemcova, Kristyna Hrdlickova, Stefani Kalli, Alina Davletova, Mario Filipe Rodrigues Saraiva Duarte, Darya Molodina, Tiina Riekki, Antonin Sebela

**Affiliations:** ^1^ Centre of Perinatal Mental Health, National Institute of Mental Health, Klecany, Czechia; ^2^ First Medical Faculty, Charles University, Prague, Czechia; ^3^ Faculty of Arts, Charles University, Prague, Czechia; ^4^ Third Medical Faculty, Charles University, Prague, Czechia; ^5^ Research Unit of Clinical Medicine, University of Oulu and Wellbeing Services County of North Ostrobothnia, Pohde, Finland

**Keywords:** perinatal mental health care, mental health policy, WHO Europe region, screening, treatment, guidelines, perinatal mental health care model, perinatal mental disorders

## Abstract

**Background:**

Although perinatal mental disorders are the most common health complication among women in the perinatal period, there is a huge gap in the implementation of related research findings in the health care system. We mapped the state of perinatal mental health (PMH) care in the WHO Europe region with aim to identify leading countries, which can serve as models for countries with less developed perinatal mental health care.

**Methods:**

Guidelines, policies, and documents related to screening and treatment services for PMH were searched as grey literature. Results were analysed to assess the status of PMH care in the WHO European countries and to identify gaps (absence of relevant service or documents). The state of perinatal mental health care was scored on a 0-5 scale.

**Results:**

The grey literature search resulted in a total of 361 websites. Seven countries (Belgium, Finland, Ireland, Netherlands, Sweden, UK, Malta) received full points for the presence of relevant PMH services or documents, while five countries received zero points. Most WHO European countries (48/53) have general mental health policies, but only 25 countries have policies specifically on perinatal mental health. Ten countries offer PMH screening, and 11 countries offer PMH service (of any type). Any PMH guidelines were provided in 23/53 countries.

**Conclusions:**

Perinatal mental health care is in its infancy in most WHO European countries. Leading countries (Belgium, Finland, Ireland, Netherlands, Sweden, UK, Malta) in PMH care can serve as conceptual models for those less developed and geopolitically close.

## 1 Introduction

Common perinatal mental disorders (CPMDs) are the most frequent complications of pregnancy, childbirth and the postpartum period with the prevalence among women at nearly 20% (1, 2). Additionally, if left untreated, CPMDs can negatively affect the well-being of mother, her baby and whole family. For instance, perinatal depression (PPD) is associated with economic hardship (3), poorer quality of life (4), relationship problems (3) or risky behavior (smoking or suicidal behavior) (5) in women. Further, it can also lead to pregnancy complications (preeclampsia, spontaneous abortion) or abnormal fetal or child development (placental abnormalities, intrauterine growth restriction, low birth weight) (6, 7). The negative impact may persist well beyond the early postnatal period as exposed children showed behavioral problems, poorer cognitive abilities, and executive dysfunction (8–10) and emotional problems (11) later in their life.

In addition, CPMDs represent a significant burden for society as treating the negative outcomes associated with untreated CPMDs in the US was estimated at $14.2 billion in 2017 (12). In the UK, the known cost is estimated at £8.1 billion per year; with 72% allocated to addressing the negative consequences associated with child morbidity (13, 14). Although mental health conditions are common, in many countries worldwide women do not receive the care they need (15–17).

The WHO developed a guide for integration of PMH in maternal and child health services, which recommends the implementation of screening services and treatment services (e.g. evidence-based interventions, pharmacotherapy) into national policies to mitigate the impact of CPMDs (18). McNab and colleagues (2022) suggested that if we want to improve the PMH care, we must prioritize women’s needs by setting global standards, guidelines, and strategies to fully integrate PMH into existing general mental health programs; and develop indicators and monitoring mechanisms to continuously assess global progress towards achieving the set goals (19). Also, they proposed that governments take responsibility for women’s PMH needs and develop specific national policies.

In addition, Reinsperger and Paul (2022) recently published a scoping review of models and pathways of the PMH care with six relevant documents from four English-speaking countries (UK, Ireland, Canada, Australia) (20). This review has described well the nature of these key English-written documents (mostly guidelines). However, as the review mentioned, some documents may be specific to local political and healthcare systems and the way health care is organized in the countries of their origin. In addition, the review did a good job defining the concept of the ‘ideal’ model in the English-speaking Global North but did not map the situation in other countries. The ideal scenario would involve each country having a national document outlining a comprehensive PMH care model tailored to its specific conditions. No search has been done to identify similar documents in other European countries.

Therefore, it is important to map the current situation in individual countries in the WHO European region which is currently unclear. Such mapping can highlight gaps in the PMH care across the countries that together make up the WHO European region across the 53, covering a vast geographical area (https://www.who.int/countries).

### 1.1 Scoping review questions and objectives

Our main review question was: ´What is the current state of the PMH care across countries forming the WHO European region?’ We aimed to map the gaps in the PMH care in these countries and to identify potential regional leaders with the most developed care.

## 2 Assessment of policy/guidelines

The study protocol was registered in the Open Science Framework (https://osf.io/hetdk). We followed the PRISMA guidelines for scoping reviews (21). For the PRISMA-ScR Checklist see **Supplementary Material 1**.

### 2.1 Search strategy

We performed a manual search of grey literature for relevant information for each country in the WHO European Region using: 1) governmental websites, 2) non-governmental websites, 3) websites of national psychiatric and gynaecological societies.

We chose to do a search of the grey literature rather than the academic literature because official screening and treatment guidelines and proclaimed national policies are generally published on government and non-government websites rather than as part of academic publications. In our review, we were not interested in research activity in individual countries.

Further, we searched for relevant information using the Google browser. We used this search code:

(‘Perinatal mental health’) AND (‘care’ OR ‘policy’ OR ‘screening’ OR ‘treatment’ OR ‘guideline’) AND (‘*the name of the country’*)

For each country, we used the English search codes and then translated them into the official language(s) of the country and performed the search again. If a country had more than one official language, we translated the search code into all of them. Thus, for each country, we used five combinations of the search code to search in English and at least five combinations in the official language of the country. After each search, we evaluated the first 50 web links, and of these 50 links, we only subjected the links relevant to the topic to further analysis. We verified that including more links would already provide irrelevant information. We then evaluated the relevant links to see if they met the eligibility criteria and created a “List of included resources” from which we drew relevant information for our results.

### 2.2 Eligibility criteria

The inclusion and exclusion criteria for relevant documents are described in **Table 1**.

**Table 1:** The inclusion and exclusion criteria for searched documents.

	Inclusion criteria	Exclusion criteria
Population	Perinatal (from the beginning of pregnancy to one year postpartum) women and their families	Women and their families outside the perinatal period
Study design	National and regional (affecting more than half of the country’s population) policy/guideline/strategy documents	-Regional (affecting less than half of the country’s population) policy/guidelines/strategy documents -Research studies -Comments
Setting	Countries of the WHO European region	Other countries than members of the WHO European region
Languages	-Czech, English, Slovak, Russian, Portuguese, Spanish, French, Greek fluently -Searches in other languages were performed using an online translator	–
Year	The document was published between 2004 and 2023	–
Search period	16/05/2022	25/09/2023

–, not applicable.

### 2.3 Selection of sources of evidence

The grey literature search in pre-selected websites (governmental websites, non-governmental websites, websites of national psychiatric and gynaecological societies) was conducted by two 4th year and two 5th year medical students (SK, AD, MD and DM) who participated in the preparation of the manuscript as research assistants. The assistants were divided into two groups, each group reviewing sources from one half of the countries in the WHO European Region. Within a group, research assistants searched and reviewed online resources independently. Final results were based on a comparison of their independent work. Collectively, the group was fluent in English, Czech, Russian, Portuguese, Spanish, Slovak, French and Greek. For European countries using languages other than these, the search was conducted using an online translator (www.deepl.com or Google Translate) to find resources in the official language of these countries. For the grey literature search, in which a search code was used, two other researchers (AH and HN) were involved. Together, they were able to read in English, Czech, Spanish and French, and searches in other languages were again conducted using the translators.

### 2.4 Data charting process and data items

The characteristics of the literature sources were captured in a pre-prepared table that included the following information: country of origin; website address; document title; date of publication; authors; main objective/topic; brief summary of content; type of policy, guidelines or plan described. Data charting was carried out independently by two researchers; in case of unresolvable discrepancies, a third researcher was involved.

### 2.5 Synthesis of results

The synthesis of the results of the online resources search was carried out for each country using the following scoring system; a country could score between 0 and 5 points, with one point awarded for each if: 1) there is a general national mental health policy; 2) there is a specific national PMH policy; 3) implemented PMH screening service is present (nationally or in more than half of the country’s population); 4) it provides any evidence-based treatment option for PMH difficulties (nationally or in more than half of the country’s population); 5) there are any evidence-based guidelines for PMH care (for diagnostics, prevention, treatment, screening or pharmacotherapy for any type of PMH problem).

Countries were ranked according to the number of points they scored. Further, we identified the WHO European leaders in PMH care and countries that lack this specialised care.

## 3 Results

### 3.1 Search results

For each of the 53 countries, we included at least 500 sources (web links) in the initial analysis (250 in English and 250 in the official language of the country), except for countries where English is the official language and where only 250 web links were included. If a country had more than one official language, we analyzed additional 250 weblinks for each additional language. Thus, approximately 26,500 links were selected. Of these 26,500, 25,230 were excluded because they did not have relevant content. This left 1,270 web links, of which only 361 provided information on relevant documents (policies, guidelines) and were included in the final analysis. The full list is shown in **Supplementary Material 2**.

### 3.2 Review findings

**Figure 1:**
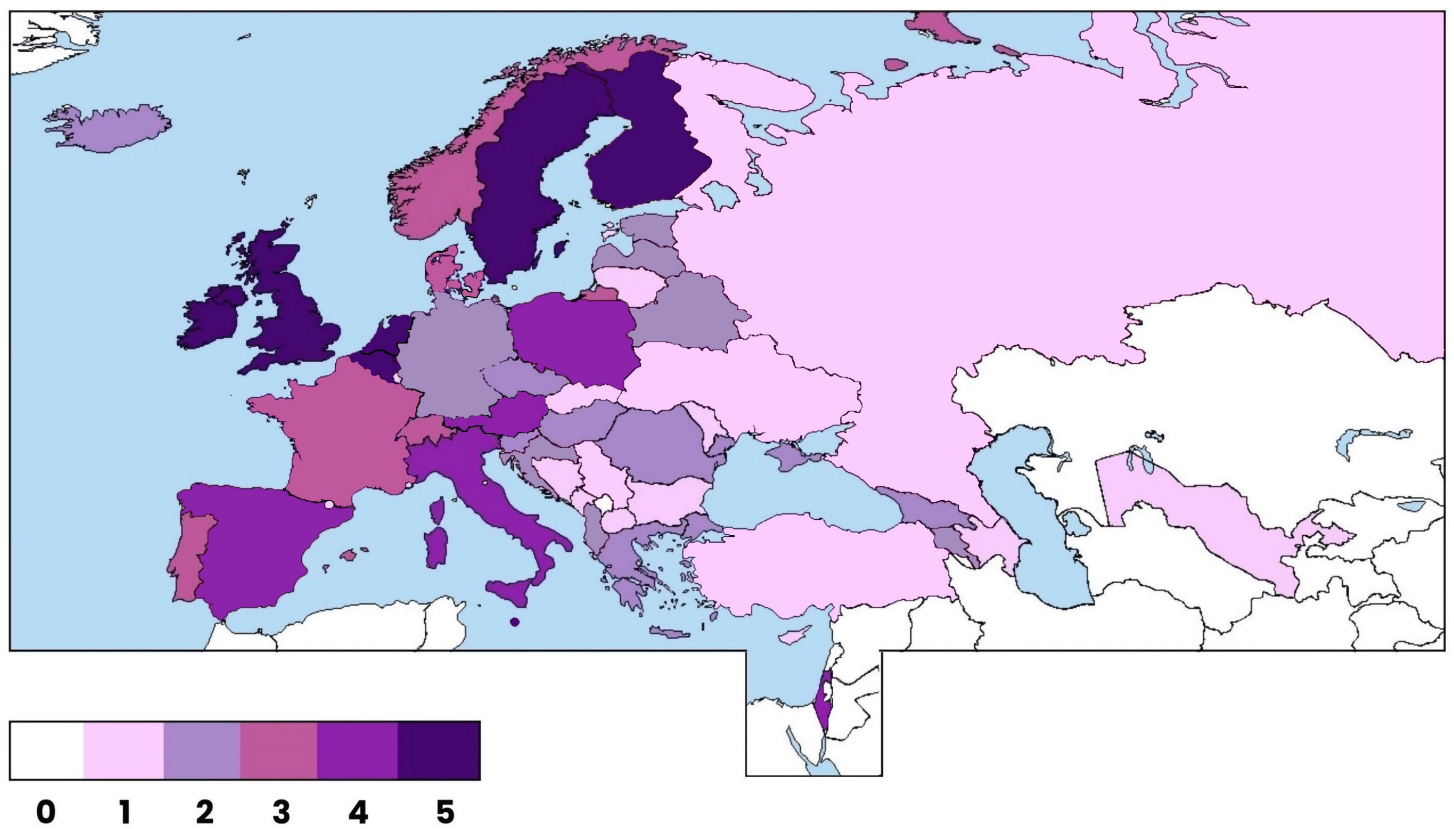
The status of perinatal mental health in the WHO-European region. The status was assessed based on a point system ranging from 0 to 5 for each country. The allocation of points reflects the level of development in perinatal mental health care, with a higher score indicating more advanced and comprehensive care.

All presented information originating from the 361 resources searched online are presented in **Table 2**; **Figure 1**.

**Table 2:** Shows the points awarded according to the presence of relevant documents describing perinatal mental health care in each country.

Country	Sum of Points	Implemented Screening program	Implemented Treatment Service	Policy mental health general	Policy specific to perinatal mental health	Guidelines
Belgium	5	1	1	1	1	1
Finland	5	1	1	1	1	1
Ireland	5	1	1	1	1	1
Netherlands	5	1	1	1	1	1
Sweden	5	1	1	1	1	1
United Kingdom	5	1	1	1	1	1
Malta	5	1	1	1	1	1
Austria	4	0	1	1	1	1
Israel	4	1	0	1	1	1
Italy	4	1	0	1	1	1
Poland	4	1	0	1	1	1
Spain	4	0	1	1	1	1
France	3	0	1	1	0	1
Denmark	3	0	0	1	1	1
Norway	3	0	0	1	1	1
Portugal	3	0	0	1	1	1
Switzerland	3	0	1	1	1	1
Estonia	2	0	0	1	1	0
Albania	2	0	0	1	0	1
Armenia	2	0	0	1	1	0
Czechia	2	0	0	1	1	0
Germany	2	0	0	1	1	0
Georgia	2	0	0	1	0	1
Greece	2	0	0	1	0	1
Hungary	2	0	0	1	0	1
Iceland	2	0	0	1	1	0
Romania	2	0	0	1	0	1
Belarus	2	0	0	1	1	0
Croatia	2	0	0	1	1	0
Latvia	2	0	0	1	0	1
Slovenia	2	0	0	1	1	0
Russian Federation	1	0	0	1	0	0
Serbia	1	0	0	1	0	0
Andorra	1	0	0	1	0	0
Azerbaijan	1	0	0	1	0	0
Bosnia and Herzegovina	1	0	0	1	0	0
Bulgaria	1	0	0	1	0	0
Cyprus	1	0	0	1	0	0
Lithuania	1	0	0	1	0	0
Luxembourg	1	0	0	1	0	0
Monaco	1	0	0	1	0	0
Montenegro	1	0	0	1	0	0
North Macedonia	1	0	0	1	0	0
Republic of Moldova	1	0	0	1	0	0
Slovakia	1	0	0	1	0	0
Uzbekistan	1	0	0	1	0	0
Turkey	1	0	0	1	0	0
Ukraine	1	0	0	1	0	0
Kazakhstan	0	0	0	0	0	0
Kyrgyzstan	0	0	0	0	0	0
San Marino	0	0	0	0	0	0
Tajikistan	0	0	0	0	0	0
Turkmenistan	0	0	0	0	0	0

Only seven countries scored the full number of points: Belgium, Finland, Ireland, Malta, Netherlands, Sweden, and United Kingdom. In contrast, five countries received zero points: Kazakhstan, Kyrgyzstan, San Marino, Tajikistan, and Turkmenistan. We found that majority of countries have a national mental health policy (48/53), but a specific perinatal mental health policy was presented in only 25 countries. Only 10 countries have established perinatal mental health screening and only 11 countries provided any type of treatment service specific to PMH. Further, less than half of the countries (23/53) have some official guidelines on any of the various aspects of PMH care (therapeutic interventions, medication options, etc.).

## 4 Actionable recommendations from leading countries

### 4.1 Belgium

Belgium has recently published a protocol on mental health care for the screening, detection and treatment of perinatal anxiety and depressive disorders, adapted to the Flemish settings (which accounts for approximately 60% of total population). The protocol is currently being implemented with the support of the Flemish Ministry of Welfare, Public Health and Family.

According to the protocol, psychosocial assessments to identify women at risk are conducted by midwives in the hospital and take approximately 30 min during a standard hospital visit at 16 weeks of gestation. Women are further screened by midwives at week 20-21 of pregnancy at the ultrasound visit using the Whooley questions and Generalized Anxiety Disorder 2-item (GAD-2), and a positive score indicate administration of the Edinburg Postnatal Depression Scale (EPDS). The Generalized Anxiety Disorder 7-item (GAD-7) may also be administrated if women score positive in the anxiety-related EPDS questions. Screening is repeated at the postnatal gynaecological visit at 6 weeks and at the routine medical check-up of the baby at 6 months. At all steps, women whose scores exceed thresholds are referred for further clinical assessment to a psychologist or psychiatrist working at the maternity services or to their GP. If a woman is diagnosed, treatment is offered directly at the maternity service unit in close collaboration with the midwives, obstetrician, and social services. There is also the option of attending the mother-baby unit or perinatal day-clinic services in severe cases.

### 4.2 Finland

According to Finish national guidelines, psychosocial risk factors should be screened and regularly monitored during prenatal visits through various standardized questions and screening tools. Further, there is an extensive health examination performed at 13-18 gestational weeks that also addresses woman’s mental health and sources of social support using the EPDS and the Beck Anxiety Inventory, and then again at 35-36 gestational weeks. After birth, maternal mood is again assessed in the first week and Postpartum depression is screened via the EPDS in week 5-12. There is close collaboration between prenatal and Child Health Services (CHS) in Finland, forming one continuous system. Perinatal psychiatric outpatient clinics are currently being established in Finland in collaboration with hospitals to strengthen care in prenatal and CHS. If mental health difficulties are identified during screening, support is provided either at 1) a prenatal clinic, 2) a mental health clinic or through 3) a family counselling centre, depending on the severity of the problem. Mild to moderate mental health problems can be treated in primary care, for example through targeted psychoeducation groups run by psychiatric nurses. For moderate problems, a psychiatrist or psychologist from secondary care would be engaged in the care team. In such cases, treatment takes place in a hospital outpatient clinic, as no specialized mental health services are provided in primary care. Finally, severe mental illnesses would be treated in specialized units in tertiary care.

### 4.3 Ireland

In 2016, Ireland developed a National Maternity Strategy (Department for Health, 2016) which brings out a plan for the development of a model of care containing PMH services. It also emphasised the need to ensure integration of services (mental health, midwifes, obstetricians, GPs and public health nurses). The care model suggests that specialist perinatal mental health services should be delivered on a ‘hub and spoke’ clinical network model: the maternity service unit with the highest number of deliveries should be the ‘hub’ (i.e. a more centralised unit with a higher level of skills and knowledge). The other, smaller units are ‘spokes’, each linked to one of the larger ‘hub’ hospitals. Each ‘hub’ should have a specialist PMH service with multidisciplinary staff, led by a consultant perinatal psychiatrist. In the ‘spokes’ units, input to the maternity service would be provided by a liaison psychiatry team supplemented by a mental health midwife. This team is linked to the ‘hub’ specialist PMH team for training, clinical advice and regular meetings.

The screening test in Ireland is usually carried out by GPs (prior to an appointment at the relevant antenatal clinic) or practice and public health nurses (at each contact with the mother and baby) or midwifes (at the booking visit); the Whooley questions are recommended. Women with milder problems should be seen by a mental health midwife (should be available at each maternity hospital/unit) and women with more severe problems by the specialist PMH team; further treatment can take place either in 1) the specialist PMH services or, in more severe cases, in 2) the mother-baby units.

PMH midwives play a key role in Ireland. A self-assessment framework document was additionally developed to support new and existing PMH midwives. PMH midwives also provide information on the local mental health support available and link women to GP. GPs link women with the professional support and counselling: Primary Care Psychology Service, Counselling in Primary Care Services (CIPC) or Local Mental Health Services, where a woman’s care plan is tailored to her personal needs. According to the Women’s Health Action Plan for Ireland 2022 – 2023, it is planned that by the year 2023 PMH services should be embedded in all maternity units.

### 4.4 Malta

Malta is a small island country with only around half a million inhabitants. In response to the Parenting National Strategy Policy 2016-2024 and influenced by the Parent-Infant Mental Health Alliance, changes in PMH care for women and their families have been introduced in Malta. Malta has a specialized Perinatal Mental Health Clinic in one of its hospitals (which is sufficient to cover the entire perinatal population of the country). Specialized psychiatric care and psychotherapy is offered there, and partners are welcomed. Women are also offered support by the midwives at Beginnings Midwifery Clinic. In addition, a routine screening has been piloted across the country, starting in 2021. The routine screening assesses the mental health of expectant parents and their relational wellbeing before and after birth. Post-pregnancy screening is carried out during a home visit by a midwife between the fifth and sixth week. Help is provided to parents who are living in an adverse situation and need professional support.

### 4.5 Netherlands

Pregnancy care in the Netherlands is mainly provided by primary care midwives who monitor not only the physical but also mental state of women and make specific referrals for treatments where needed. For monitoring midwives use standardised online questionnaires, which are provided and validated by the National Knowledge Centre for Psychiatry and Pregnancy (established in 2009 to improve mental healthcare in the perinatal period). The screening tool includes questions on mental health and psychosocial problems and the EPDS. Midwives are also trained on how to support women and how to recognize severer mental health problems. After delivery, the mother’s mental health is monitored at the CHS. Dutch hospitals and outpatient clinics offer PMH services by the so-called ‘POP care’ team (psychiatry, obstetrics and paediatrics). The multidisciplinary POP team usually additionally includes other professionals such as psychologists, midwifes, social workers, etc. The team offers various treatment options, including preconception counselling, psychological treatment (e.g. cognitive-behavioural therapy, eye-movement desensitisation and reprocessing) or pharmacological treatment. There are also specialised medical-psychiatric departments in hospitals for women with severe psychiatric diagnosis (e.g. mother-baby units). National guidelines are available for health professionals to achieve the high standards for pharmacological treatment.

### 4.6 Sweden

In Sweden, antenatal and CHS are part of the primary care system in every region, are free of charge, and almost 100% of pregnant women and their families attend regular check-ups (usually 8-11 times), providing the Swedish system with good conditions for optimising PMH care. In addition, universal comprehensive and well-established screening programs are provided for all women in the perinatal period (with a closer focus on those with existing mental illness or fear of childbirth and/or parenthood). Screening is coupled with follow-up care, which includes regular midwife visits up to 16 weeks postpartum or person-centred counselling (‘listening visits’) provided by CHS nurses to women who score above the threshold on the EPDS 6-8 weeks postpartum. Guidelines from the Swedish Society of Obstetrics and Gynaecology offer detailed instructions (e.g., the threshold for the Swedish version of the EPDS).

### 4.7 United Kingdom

The National Institute for Health and Care Excellence (NICE) is the official regulatory authority for improving PMH care in the UK. In 2014, NICE published official clinical guidelines for various healthcare professionals and commissioners (last updated 2020) on the recognition, assessment, and treatment of mental health problems in women in the perinatal period. It covers a variety of conditions and promotes their early detection and management. It suggests that a woman should be asked about her mental health and wellbeing at her first contact with primary care at the booking visit and in the early postnatal period with either the Whooley questions or using the GAD-2. If a woman answers positively to these questions, the EPDS or Patient Health Questionnaire (PHQ-9) should be used for a full assessment. If testing is again positive, the woman should be referred to a GP or, if a severe mental health problem is suspected, directly to a mental health professional.

The NHS England perinatal programme aims to ensure that there is a dedicated community PMH team in every area in England offering psychiatric and psychological assessments and other care for women. There are also several national support groups that can be contacted for advice: Association for Post Natal Illness (APNI); Pre and Postnatal Depression Advice and Support (PANDAS). For more severe cases, there are well-established inpatient mother-baby units in the UK, commissioned by NHS England. Community and inpatient teams are multidisciplinary and have strong links with the maternity services, health visiting, primary care and social care.

## 5 Discussion

Our scoping review brings a closer look at the current state of perinatal mental health (PMH) care in the WHO Europe Region. We reviewed available grey literature on the presence of documents and services related to women’s mental health care in the perinatal period in each country. We looked at whether countries had a national mental health policy in general and a mental health policy specific to the perinatal period. We also looked at whether countries had official guidelines on any PMH issue (prevention, diagnosis, treatment). Further, we assessed whether there was a screening program available in the country, provided nationally or in at least a region of the country of more than 50% of the total population. In the same way, we reviewed at whether the country had any treatment services available specific to PMH pathology. We used these indicators for the final assessment of the status of PMH care in each state and then ranked the states according to their score.

Countries can be listed according to their Gross National Income (GNI). Importantly, all the PMH care leaders (countries who scored higher in our review: Belgium, Finland, Netherlands, Malta, Sweden, UK) are countries with higher income (GNI per capita of $13,206 or more); these countries might serve as a positive model for some geopolitically close countries with also high income to develop their own PMH care systems.

However, the development of health care system in countries does not depend only on the relative income of the country. The results of our evaluation show that Western European countries (Belgium, Finland, Netherlands, Sweden, UK) are ahead of Central and Eastern European countries in the development of PMH care. Differences between global East and West might be explained by differences in the current institutional arrangements of health care systems in the two regions; i.e. lower financial resources, higher out-of-pocket payments and lower supply of primary health care services in Eastern European countries compared to Western European countries (22). More important for citizens’ evaluation are the policies that determine the financial aspects of health care provision - the amount of resources invested in health care, the priority given by the government to health care, and the way the public-private mix is managed and the supply of health personnel in primary care (21).

Sadeniemi and colleagues (2018) go on to compare mental health services (MHS) in two Western European countries, one located in northern and the other in southern Europe. They cite Finland, which scored full points (5) in our review, as representative of the European North, and Spain, scoring 4 points in our review, as representative of the European South. They found out that there are 6.7 times more staff resources at the MHS in Finland than in Spain and better resource allocation. Thus, even in the European context significant differences exist in the structure and resourcing of MHS (23).

One non-Western country that received full points (5) was Malta, an island nation with a population of just over half a million. Malta is a very small country, so one hospital providing specialised perinatal care has the capacity to cover the care of the perinatal population in the whole country. This means that in other smaller European countries, specialised care may be similarly concentrated in just one centre (e.g. a large hospital). Poland scored highest in the Central Europe, Estonia and Latvia scored 2 points to lead the Baltics, countries in the Southern Europe received between 1-2 points. The Central Asian countries (Kazakhstan, Kyrgyzstan, Tajikistan, Turkmenistan, Uzbekistan), which are also considered part of the geopolitical configuration of the WHO European Region (also having the lowest GNI per Capita in this region), were at the lower end of the overall ranking.

According to our review, most of the countries (Bulgaria, Estonia, Slovakia, to name just a few) have not introduced any official PMH policy yet, while general mental health policies have been introduced in most countries (48/53). Some countries have introduced screening or treatment programmes (e.g. Austria, Ireland, Netherlands), some of which are government-supervised (e.g. UK, Finland, Portugal). Countries with progressive PMH care usually offer guidelines with steps on how to identify women at risk and how to follow up (Austria, Sweden, Netherlands, UK); these guidelines are needed for a consistent and professional approach by care providers within the country and their implementation should be enforced by the authorities. Some countries (e.g. Poland) have implemented a screening service, but at the same time they lack implementation of a specialized treatment service that could be universally offered to women identified at risk, which may lead to a gap in care delivery.

Further, countries with high activity have developed perinatal crisis networks, which are usually multidisciplinary and offer comprehensive care (Austria, UK); have introduced midwives or lay home visits (Sweden, UK) and various national support groups; and have introduced specialist services such as perinatal centres and mother-baby units in the UK. The development of PMH care in European countries should be influenced by a regulatory body that can directly influence national policies, in the UK this is the National Institute for Health and Care Excellence (NICE) and in the Netherlands, it is the National Knowledge Centre for Psychiatry and Pregnancy.

## 6 Study limitations

Our findings must be interpreted in the context of several limitations.

The findings of our review may have been affected by the language proficiency barrier. Together the research team was able to speak eight different languages and used online translators to search for relevant material written in other languages which might have affected quality of the overall search and translation process because documents such as national guidelines are usually written in the native languages.

Some countries may not have published the relevant documents for public use and access to them may be limited. However, because we did not analyse the quality of the included documents and focused only on their existence, which we scored as either 1 - document present in the country or 0 - document absent in the country, we believe that our results provide an approximate model of the current situation of PMH care in the WHO European region.

WHO recommends the implementation of systematic and universally provided screening according to its guidelines (18). For this reason, we awarded a point for a screening program or treatment service implemented if it has been implemented at a national level. But some of the countries mapped have more than one official language (e.g. Belgium or Switzerland) and some are divided into several autonomous units which may have different health policies (e.g. Germany). In these cases, the application of national programs may face obstacles or may even be impossible. Therefore, in order not to disadvantage these countries in the analysis, we have not only given a score for national programs, but also for regional programs that has been implemented in more than 50% of country’s total population.

However, in our analysis, we encountered the problem of distinguishing the level at which services were provided, because for some documents the guarantor of these programs/services was not specified. As a result, we were sometimes unable to distinguish whether the project was national or local and what proportion of population it covers. In cases where the guarantor could not be traced, the country scored zero, which may not reflect reality. Therefore, the absence of points for implemented screening and treatment services does not mean that a country does not actually provide these services in some regions of the country (as in case of Germany). On the other hand, Belgium is an example of a country that received a point for an implemented screening program, although it was not a national program. In Belgium there is a screening program only for the Dutch-speaking (Flanders) region of the country with 57% of the total population, but this population was sufficient to obtain a point.

We reviewed the presence of relevant documents in the countries. But we are also aware that the presence of a document describing the implementation of a screening program or treatment service does not mean that it is applied and followed in practice. The treatment gap in PMH care remains significant, despite recent developments in this area (15–17).

Lastly, we also originally planned to collect information on the state of PMH care in each country from selected national experts (see our study protocol at https://osf.io/hetdk), whom we contacted by email, to verify and complete our analysis of the searches. The national expert survey consisted of several open and closed questions describing the presence of specialized guidelines/policies and services in each country. However, as the response rate was only 23% (12/53), we decided to exclude the collected data from analysis.

## 7 Future directions

Mental health care for women and their families is becoming a very important topic globally and is reflected in the intensity of research activity in this field. However, most countries still lag behind with the implementation of PMH care. In other countries, care is already implemented, although evidence of its (cost)effectiveness is often lacking.

In this context, Manolova and colleagues (2023) talk about the concept of “voltage drop”, where a program with high efficacy may not achieve high effectiveness in the real world (24). Studies evaluating (cost)effectiveness and feasibility of existing interventions and programmes are both important for care development and often lacking. Moreover, dissemination of findings from such studies could lead to the development and adaptation of PMH care and help to strengthen and streamline delivery both within and across countries.

However, universal adaptation of PMH care is not possible and one should always look at the cultural aspects of particular groups in unique countries, regardless of the economic income of the country. Thus, existing policies, resources, local needs, and the demand for PMH care need to be taken into account when implementing care, otherwise care may be ineffective. Research can help to identify barriers in specific settings (23).

Notably, according to the WHO guidelines (2020), the common starting point for the implementation of care in individual countries, regardless of cultural and geopolitical conditions, is the integration of PMH care into primary care, i.e. the training and involvement of non-specific health providers in the providing of mental health care, which should take place within the stepped care model (18).

We recognize the limitations of a universal care delivery system, and therefore the model that results from our scoping review is not intended to be a substitute for guidance on the development of PMH care in individual countries, but only to map the situation and highlight countries where a model of care already exists.

There is a need to appeal to authorities in individual countries to see care of women in the perinatal period as a priority for revision of routine physically oriented care for women in this period in primary care settings.

## 8 Conclusion

Perinatal mental health care is at an early stage of development in most WHO European countries. The current situation in the perinatal care system generally reflects the overall trend of countries towards innovation in mental health care more broadly. Leading countries (Belgium, Finland, Ireland, Netherlands, Sweden, UK, Malta) in PMH care can serve as conceptual models for those less developed and geopolitically close.

## Author contributions

AH: Writing – original draft, Project administration, Investigation, Formal analysis, Data curation, Conceptualization. HN: Writing – review & editing, Investigation, Formal analysis. KH: Writing – review & editing, Investigation. SK: Writing – review & editing, Investigation. AD: Writing – review & editing, Investigation. MD: Writing – review & editing, Investigation. DM: Writing – review & editing, Investigation. TR: Writing – review & editing. AS: Writing – review & editing, Supervision, Methodology, Conceptualization.
