# Supplementary material for: State of perinatal mental health care in the WHO region of Europe: a scoping review
Source: Front Psychiatry. 2024 Mar 13;15:1350036. doi: 10.3389/fpsyt.2024.1350036 (PMC10965802; doi:10.3389/fpsyt.2024.1350036)
Supplement: Supplementary file 1 [file DataSheet_1.docx]

List of Included Resources

Albania

<https://www.ditenate.al/blog/depresioni-pas-lindjes-post-partum>

<https://shendetesia.gov.al/wp-content/uploads/2018/02/Paketa_e_rishikuar_e_miratuar.pdf>

<https://extranet.who.int/countryplanningcycles/sites/default/files/planning_cycle_repository/albania/draft_strategt_albania_2016-2020.pdf>

<https://www.mindbank.info/collection/country>

<http://qkev.gov.al/images/Draft_Strategjia_30Maj2016_web.pdf>

<https://assets.publishing.service.gov.uk/government/uploads/system/uploads/attachment_data/file/1105170/ALB_CIN_Mental_Health.pdf>

<https://www.ishp.gov.al/wp-content/uploads/2017/05/PLANI-VEPRIMIT-PROM-SHEND_2017-2021.pdf>

Andorra

<https://www.salut.ad/images/stories/Salut/pdfs/temes_salut/PISMA.pdf>

Armenia

<https://www.moh.am/#1/900002>

<https://www.moh.am/images/legal-889.pdf>

<http://www.aaog.am/index.html>

<https://www.apnet.am/arm/7/%D4%BB%D6%80%D5%A1%D5%BE%D5%A1%D5%AF%D5%A1%D5%B6-%D5%A1%D5%AF%D5%BF%D5%A5%D6%80.html>

<https://www.moh.am/uploads/N%2015.pdf>

<http://www.irtek.am/views/act.aspx?aid=91794>

<https://www.arlis.am/DocumentView.aspx?docid=156362>

<https://www.arlis.am/documentview.aspx?docID=89318>

Austria

<https://www.wien.gv.at/gesundheit/beratung-vorsorge/frauen/frauengesundheit/schwerpunkte/lebensphasen/schwangerschaft/perinatal.html#:~:text=Sie%20betrifft%2015%20bis%2020,und%20deshalb%20oft%20unerkannt%20bleibt>

<https://www.mindbank.info/item/5514>

<https://www.mindbank.info/item/4034>

<https://www.sozialministerium.at/Themen/Gesundheit/Eltern-und-Kind/Frühe-Hilfen.html>

<https://www.fruehehilfen.at/fxdata/fruehehilfen/prod/media/downloads/Berichte/NZFHat_Development-and-Implementation-of-Early-Childhood-Interventions-in-Austria_Feb_2019.pdf>

https://eprints.aihta.at/1437/1/HTA-Projektbericht_Nr.151.pdf

Azerbijan

[http://egip-az.com](http://egip-az.com/)

<https://sehiyye.gov.az/>

<http://www.psychiatry.az/>

<http://www.psm.az/uploads/faydali_melumatlar/Psixi_saglamliq_sahesinde_milli_strategiya.pdf>

<https://azerbaijan.unfpa.org/sites/default/files/pub-pdf/A4_az_0.pdf>

Belarus

<https://www.medcenter.by/documents-downloads/publicacii/akusherstvo-ginekologiya/sbornik11012021.pdf>

<https://gs.archives.gov.by/?page_id=7879>

<http://healthcare.by/instinfo.php?orgnum=12875>

<http://minzdrav.gov.by/>

<http://16gdp.by/informatsiya/pamyatki/893-zashchita-materinstva-i-detstva-v-respublike-belarus>

<https://mentalhealth.by/media/doc/protocol.pd>

Belgium

<https://www.thevillage.be/parenting/postnatal-support/>

<https://www.domusmedica.be/actueel/niet-elke-zwangerschap-verloopt-rooskleurig#:~:text=Eén%20moeder%20op%20vijf%20heeft,kampen%20met%20een%20postnatale%20depressie>

<https://www.uzgent.be/patient/zoek-een-arts-of-dienst/psychiatrie/psychiatrisch-zorgaanbod/perinatale-mentale-gezondheid>

<https://www.kindengezin.be/nl/professionelen/over>

<https://kce.fgov.be/fr/depistage-de-la-depression-pendant-et-apres-la-grossesse>

<https://kce.fgov.be/sites/default/files/2021-11/KCE_318_Mental_Health_care_Report_0.pdf>

Bosnia and Herzegovina

<https://udruzenje-ginekologa.ba/sekcija-za-perinatalnu-medicinu/>

<http://www.fmoh.gov.ba/index.php/zakoni-i-strategije/strategije-i-politike>

<http://www.fmoh.gov.ba/images/federalno_ministarstvo_zdravstva/zakoni_i_strategije/strategije_i_politike/dokumenti/Politika_i_Strategija_mentalno%20zdravlje_FBiH.pdf>

<https://upubih.com/dokumenti/>

[https://ba.unfpa.org/sites/default/files/pub pdf/ljudska_prava_u_oblasti_seksualnog_i_reproduktivnog_zdravlja_u_bih.pdf](https://ba.unfpa.org/sites/default/files/pub%20pdf/ljudska_prava_u_oblasti_seksualnog_i_reproduktivnog_zdravlja_u_bih.pdf)

<https://www.zzjzfbih.ba/wp-content/uploads/2019/02/2016.pdf>

Bulgaria

<https://bsobgyn.com/gaidlaini/>

<http://bsobgyn.com/guidelines/akusherstvo/16.%20%D0%9F%D0%BE%D1%81%D0%BB%D0%B5%D1%80%D0%BE%D0%B4%D0%BE%D0%B2%20%D0%BF%D0%B5%D1%80%D0%B8%D0%BE%D0%B4.pdf>

<https://www.mh.government.bg/bg/politiki/strategii-i-kontseptsii/strategii/nacionalna-strategiya-za-psihichno-zdrave-na-grazhdanite-na-repu/>

<https://www.mh.government.bg/bg/politiki/strategii-i-kontseptsii/strategii/nacionalna-strategiya-za-psihichno-zdrave-na-grazhdanite-na-repu/>

<https://www.mh.government.bg/media/filer_public/2021/04/14/programa-majchino-detsko_zdrave-21-30.pdf>

<https://www.parliament.bg/pub/cW/20151120030041NZS-2020.pdf>

Croatia

<https://www.psihijatrija.hr/site/category/smjernice/>

<https://www.psihijatrija.hr/site/wp-content/uploads/2019/11/HRVATSKE-SMJERNICE-ZA-LIJE%C4%8CENJE-SHIZOFRENIJE-I-DRUGIH-PSIHOTI%C4%8CNIH-POREME%C4%86AJA.pdf>

<https://www.psihijatrija.hr/site/wp-content/uploads/2020/03/SMJERNICE-DEPRESIJA-2020-s-izmjenama.pdf>

<https://vlada.gov.hr/UserDocsImages/2016/Sjednice/Arhiva/79-4.pdf>

<https://esavjetovanja.gov.hr/ECon/MainScreen?entityId=20208>

<https://zdravstvo.gov.hr/UserDocsImages//2022%20Objave//Nacionalni%20plan%20razvoja%20zdravstva%202021.-2027..pdf>

Cyprus

<https://panosplatritis.health/επιλόχεια-κατάθλιψη-ποιος-ο-ρόλος-της/>

<https://www.moh.gov.cy/Moh/MOH.nsf/index_gr/index_gr?OpenDocument>

<https://shso.org.cy/en/patient-and-visitor/iperisies-psixikis-igeias/>

Czechia

<https://www.mzcr.cz/>

<https://www.mzcr.cz/wp-content/uploads/2020/01/N%C3%A1rodn%C3%AD-ak%C4%8Dn%C3%AD-pl%C3%A1n-pro-du%C5%A1evn%C3%AD-zdrav%C3%AD-2020-2030.pdf>

<https://www.psychiatrie.cz/reforma>

<https://postupy-pece.psychiatrie.cz/images/pdf/Stabilizacni-udrzovaci-lecba-schizofrenie-22.pdf>

<http://www.cgps.cz/>

<https://en.csupg.cz/>

Denmark

<https://econ.au.dk/the-national-centre-for-register-based-research/research/ncrr-research/hope-postpartum-depression>

<https://postpartumprogress.com/postpartum-depression-danish-public-health-experts-recommend-ppd-screening-for-all-new-moms>

<https://www.sst.dk/en/English/publications/2016/National-clinical-guideline-for-the-non-pharmacological-treatment-of-unipolar-depression>

<https://psy.ku.dk/abc/?pure=da%2Fpublications%2Fproject-newborn--preparation-for-birth-and-parenthood(e9694835-2900-4d8d-8064-d979b9482cdd).html>

<https://www.sst.dk/-/media/Udgivelser/2021/Graviditet/Gravides-mentale-sundhed/SST-Rapport_-Betydning-af-angst_TILG.ashx?la=da&hash=630D76E533A837A9D2A2DBB20E2C087FE79F2037>

<http://sundhedsformidling.dk/projekter/sammen-om-mental-sundhed.aspx>

<https://mentalsundhed.dk>

<https://www.sundhed.dk/sundhedsfaglig/information-til-praksis/nordjylland/almen-praksis/patientforloeb/forloebsbeskrivelser/p-psykisk/patientens-team-gravide-med-psykisk-sygdom/#:~:text=Medicinrådgivning%20til%20praktiserende%20læger,medicinrådgivning%2C%20gerne%20via%20et%20korrespondancenotat>.

<https://psykiatri.rn.dk/for-sundhedsfaglige/samarbejde-og-projekter/samarbejde-om-gravide-med-psykisk-sygdom/-/media/Hospitaler/Psykiatrien/For-sundhedsfaglige/Samarbejde-og-projekter/Rapport-Samarbejde-om-gravide-med-psykisk-lidelse.ashx>

Estonia

<https://www.sm.ee/et>

<https://www.sm.ee/sites/default/files/content-editors/Tervishoid/rta_05.05.pdf>

<https://www.sm.ee/et/psuhholoogi-palgatoetus-ja-vaimse-tervise-teenusetoetus>

<https://www.sm.ee/sites/default/files/vt_teenuse_toetus.pdf>

<https://psy.ee/materjalid/ravijuhised/>

<https://www.ens.ee/en/>

<http://heakool.ee/wp-content/uploads/2016/04/Vaimse_tervise_heaolu_strateegia_2016-2025_30.03.2016.pdf>

<https://ammaemand.org/wp-content/uploads/2021/12/Ämmaemanduse-arengukava_2019-2024.pdf>

Finland

<https://www.hel.fi/sote/perheentuki-en/pregnancy-and-childbirth/after-childbirth/postpartum-depression/>

<https://www.hel.fi/helsinki/en/administration/administration/services/service-description?id=3214>

<https://www.terveyskirjasto.fi/dlk00381>

<https://mieli.fi/en/>

<https://stm.fi/en/mental-health-policy-guidelines>

<https://julkaisut.valtioneuvosto.fi/bitstream/handle/10024/162234/STM_2020_15.pdf?sequence=1&isAllowed=y>

<https://www.theseus.fi/bitstream/handle/10024/511560/Opinnaytetyo_Pesonen_Tanja.pdf?sequence=2&isAllowed=y>

<https://www.kaypahoito.fi>

France

<https://www.europeristat.com/images/doc/NPHR/French_National_Perinatal_Survey_2016.pdf>

<https://www.expatica.com/fr/healthcare/healthcare-basics/a-guide-to-the-french-healthcare-system-101166/#:~:text=France%20has%20a%20high%20quality,%2C%20hospitals%2C%20and%20specialist%20providers>

<https://www.maman-blues.fr>

<https://www.gynger.fr/une-alliance-francophone-pour-la-sante-mentale-perinatale/>

<https://www.santementale.fr/2020/12/psychiatrie-perinatale-un-parcours-de-soins-ultra-precoces/>

<https://www.legifrance.gouv.fr/codes/section_lc/LEGITEXT000006072665/LEGISCTA000006171193/>

<https://www.santepubliquefrance.fr/docs/unites-d-hospitalisation-conjointe-mere-bebe-une-reponse-adaptee-un-dispositif-a-deployer>

<https://alliance-psyperinat.org/2023/04/23/commission-nationale-de-la-psychiatrie/>

Georgia

<https://www.moh.gov.ge/ka/guidelines/>

<https://www.moh.gov.ge/ka/706/>

<https://www.moh.gov.ge/uploads/files/2019/Failebi/18.04.2019.pdf>

<https://www.moh.gov.ge/uploads/files/oldMoh/2016/perinatalregion/11.pdf>

<https://www.facebook.com/gogpa/> <https://matsne.gov.ge/ka/document/view/24178?publication=17>

<https://nha.gov.ge/ge/c/fsiqikuri-janmrteloba>

<https://matsne.gov.ge/ka/document/view/3825285?publication=0>

<https://matsne.gov.ge/ka/document/view/5357283?publication=0>

<https://matsne.gov.ge/ka/document/view/2667876?publication=0>

Germany

<https://www.berliner-hebammenverband.de/en/parents/special-situations/postpartum-depression.html>

<https://schatten-und-licht.de/association/?lang=en>

<https://www.hilfetelefon-schwangere.de>

<https://www.familienzelt-berlin.de/ueber-uns/beratungsstelle/>

<https://www.apotheken-umschau.de/familie/schwangerschaft/untersuchungen/welche-untersuchungen-es-in-der-schwangerschaft-gibt-790299.html#:~:text=Nach%20den%20Mutterschafts%2DRichtlinien%20sind,Abständen%2C%20ungefähr%20alle%20zwei%20Tage>.

<https://www.familienplanung.de/fileadmin/user_upload/familienplanung.de/Checklisten/fampl_checkliste_Organisatorisches-in-Schwangerschaft.pdf>

<https://www.bundesgesundheitsministerium.de/fileadmin/Dateien/5_Publikationen/Gesundheit/Broschueren/Nationales_Gesundheitsziel_Gesundheit_rund_um_die_Geburt.pdf>

<https://www.fruehehilfen.de/fileadmin/user_upload/fruehehilfen.de/pdf/Publikation-NZFH-Handreichung-9-Eltern-psychische-Erkrankungen.pdf>

<https://marce-gesellschaft.de/materialien/>

<https://www.bundesgesundheitsministerium.de/fileadmin/Dateien/5_Publikationen/Gesundheit/Broschueren/Nationales_Gesundheitsziel_Gesundheit_rund_um_die_Geburt.pdf> ,

https://www.pcmch.on.ca/wp-content/uploads/PCMCH- Care-Pathway-for-the-Management-of-Perinatal-Mental-Health_23July2021.pdf.

<https://register.awmf.org/de/leitlinien/detail/051-032>

<https://www.bundesgesundheitsministerium.de/themen/gesundheitswesen/gesundheitsziele.html> –

Greece

<https://www.mighealthcare.eu/resources/handbooks/MIG_HEALTHCARE_155x230_GR_F01.pdf>

<https://tvxs.gr/news/ygeia/mitrotita-kai-psyxiki-diataraxi>

<https://www.fainareti.gr/en/programs/perinatal-mental-health-national-campaign>

<http://www.opengov.gr/yyka/?p=3940&cpage=1>

Hungary

<https://kollegium.aeek.hu/Iranyelvek/Index>

<https://kollegium.aeek.hu/Download/Download/2305>

<http://www.mptpszichiatria.hu/info.aspx?sp=1>

<http://mptpszichiatria.hu/hirek.aspx?&nid=87050&cid=32#87050>

<https://mnt.olo.hu/>

<https://www.origo.hu/itthon/20200212-a-mentalfor-csoporta-kuruzslotorveny-felulvizsgalatara-keri-a-jogalkotoktol.html>

<https://mok.hu/public/media/source/Transzparencia/Allasfoglalasok/Eg%C3%A9szs%C3%A9ges%20Magyarorsz%C3%A1g%202021%E2%88%922027%20Eg%C3%A9szs%C3%A9g%C3%BCgyi%20%C3%81gazati%20Strat%C3%A9gia.pdf>

<https://www.hbcs.hu/uploads/jogszabaly/2485/fajlok/EEMI_szakmai_iranyelve_a_pre_peri.pdf>

Iceland

<https://island.is/en/pregnancy-wellbeing-wersonal-circumstances>

<https://opinvisindi.is/bitstream/handle/20.500.11815/1214/Doktorsverkefni%20SSJ.pdf?sequence=1&isAllowed=y>

<https://www.althingi.is/altext/141/s/0604.html>

<https://throunarmidstod.is/svid-thih/ung-og-smabarnavernd/heimavitjanir/#Tab4>

Ireland

<https://www2.hse.ie/conditions/mental-health/postnatal-depression/screening-treatment.html>

<https://www2.hse.ie/conditions/mental-health/postnatal-depression/getting-help.html>

<https://www2.hse.ie/conditions/mental-health/postnatal-depression/advice-for-partners-and-families.html>

<https://www2.hse.ie/conditions/mental-health/postnatal-depression/postpartum-psychosis.html>

<https://www.cuidiu.ie/userfiles/file/Postnatal_Depression_A_Guide_for_Mothers%2C_Family_and_Friends.pdf>

<http://www.pnd.ie/about>

<https://www.parentline.ie/>

[ac61fd2b66164349a1547110d4b0003f.pdf (assets.gov.ie)](https://assets.gov.ie/18835/ac61fd2b66164349a1547110d4b0003f.pdf)

[gov.ie - Women's Health Action Plan 2022 – 2023 (www.gov.ie)](https://www.gov.ie/en/publication/232af-womens-health-action-plan-2022-2023/)

<https://www.hse.ie/eng/services/list/4/mental-health-services/specialist-perinatal-mental-health/specialist-perinatal-mental-health-services-model-of-care-2017.pdf>

Italy

<https://www.figo.org/societa-italiana-di-ginecologia-e-ostetricia-sigo>

<https://www.internationalinsurance.com/health/systems/italy.php>

<https://www.salute.gov.it/imgs/C_17_notizie_3378_listaFile_itemName_11_file.pdf>

<https://www.salute.gov.it/imgs/C_17_pubblicazioni_3120_allegato.pdf>

<https://www.harmoniamentis.it/disturbi-neurocomportamentali/i-sintomi-della-depressione-post-partum-da-non-trascurare/>

[Realizzazione di un intervento per il riconoscimento del disagio psichico perinatale e sostegno alla maternità fragile nei servizi del percorso nascita della AUSL di Bologna (iss.it)](https://www.epicentro.iss.it/ben/2019/novembre/disagio-psichico-perinatale-ausl-bologna)

<https://www.ncbi.nlm.nih.gov/pmc/articles/PMC8277529/#:~:text=Perinatal%20mental%20health%20service%20in%20Italy&text=Maternal%20care%20services%20are%20provided,more%20than%201800%20national%20FCCs>.

https://fondazioneonda.it/ondauploads/2014/12/Good-clinical-practice.pdf

Israel

<https://www.gov.il/he/departments/policies/bz03-2014>

<https://www.gov.il/BlobFolder/policy/bz03-2014/he/files_circulars_bz_bz03_2014.pdf>

<https://gynecology.mednet.co.il/>

<https://psychiatry.doctorsonly.co.il/>

<http://www.health.gov.il/>

<https://www.ispraisrael.org.il/sites/ispra/UserContent/files/%D7%9E%D7%93%D7%99%D7%A0%D7%99%D7%95%D7%AA%20%D7%91%D7%91%D7%A8%D7%99%D7%90%D7%95%D7%AA%20%D7%94%D7%A0%D7%A4%D7%A9/%D7%A0%D7%99%D7%94%D7%95%D7%9C%20%D7%A2%D7%A6%D7%9E%D7%99%20%D7%95%D7%94%D7%9B%D7%95%D7%95%D7%A0%D7%94%20%D7%A2%D7%A6%D7%9E%D7%99%D7%AA/11_sigal%20mautner%20(1)FINAL.pdf>

<https://www.health.gov.il/Subjects/pregnancy/Childbirth/birthday/Pages/postnatal_depressions.aspx>

https://www.nitza.org/en/contact/

Kazakhstan

<https://www.gov.kz/memleket/entities/dsm/documents/1?lang=ru>

<https://www.gov.kz/memleket/entities/dsm/documents/details/adilet/V1600013404?lang=ru>

<https://www.gov.kz/memleket/entities/dsm/documents/details/216111?lang=ru>

<https://karm.kz/arhiv-kongressov-karm/>

<https://krmu.edu.kz/kafedra-psihiatrii-narkologii-i-nevrologii/>

<https://krmu.edu.kz/en/department-of-obstetrics-and-gynecology/>

<https://adilet.zan.kz/kaz/docs/P990000999>

<https://adilet.zan.kz/eng/docs/K2000000360>

Kyrgyzstan

<http://med.gov.kg/ru/>

<http://med.kg/images/MyFiles/zakony/zakon_ob_ohrane_zdorovye_grajdan_KR.doc>

<https://www.mental-health-congress.ru/ru/news/kyrgyzskaja-psihiatricheskaja-associacija--kpa-primet-uchastie-v-kongresse/>

<https://www.osoo.kg/inn/02410200010141/>

<https://www.kgma.kg/ru/departments/clinical-departments/department-of-medical-psychology-psychiatry-and-narcology>

<http://cbd.minjust.gov.kg/act/view/ru-ru/11840>

<http://cbd.minjust.gov.kg/act/view/ru-ru/1602>

<https://tbinternet.ohchr.org/Treaties/CESCR/Shared%20Documents/KGZ/INT_CESCR_ICO_KGZ_18501_R.docx>

Latvia

<https://www.ginasoc.lv/kliniskas-rekomendacijas-2>

<https://www.ginasoc.lv/uploads/content/Dzemdibu%20palidziba/9789241549356-eng.pdf>

<https://www.vmnvd.gov.lv/lv/darbibas-plans>

<https://www.vmnvd.gov.lv/lv/media/8286/download>

<https://likumi.lv/doc.php?id=140695>

<https://pkc.gov.lv/sites/default/files/inline-files/NAP2027__ENG_2.pdf>

<https://latvija.lv/en/DzivesSituacijas/gimene/Berna-piedzimsana#show1>

Lithuania

<https://lagd.lt/specialistams/metodikos/205>

<https://lagd.lt/data/public/uploads/2020/12/akuserine-metodika_antenataline-prieziura-2020.pdf>

<https://sam.lrv.lt/lt/administracine-informacija/planavimo-dokumentai/metiniai-veiklos-planai>

<https://sam.lrv.lt/uploads/sam/documents/files/Administracine_informacija/Planavimo_dokumentai/Metiniai_veiklos_planai/SAM%202022%20MVP.pdf>

<https://www.psichiatrija.lt/naujienos/>

<https://sam.lrv.lt/lt/>

<https://sam.lrv.lt/uploads/sam/documents/files/Psichikos%20sveikatos%20forumas%202020-ataskaita(1).pdf>

<https://sam.lrv.lt/lt/veiklos-sritys/asmens-sveikatos-prieziura/diagnostikos-gydymo-metodikos-ir-rekomendacijos/diagnostikos-ir-gydymo-metodikos>

<https://e-seimas.lrs.lt/portal/legalAct/en/TAD/TAIS.39589>

Luxembourg

<https://www.expatica.com/lu/healthcare/healthcare-basics/the-healthcare-system-in-luxembourg-105466/>

<https://msan.gouvernement.lu/fr/actualites.gouvernement%2Bfr%2Bactualites%2Btoutes_actualites%2Barticles%2B2013%2B09-septembre%2B11-sante-mere-enfant.html>

<https://legilux.public.lu/eli/etat/leg/loi/2014/12/17/n2/jo>

<https://www.passage.lu/special-needs/postnatal-depression/>

<https://www.sages-femmes.lu/>

<https://sante.public.lu/fr/publications/p/plan-national-sante-mentale.html>

Malta

<https://www.welcome-center-malta.com/the-maltese-health-care-system-explained/>

<https://www.islandbebe.com/about-us/>

<https://www.islandbebe.com/expert-advice-malta/mental-health-psychology/postpartum-depression-and-postpartum-psychosis/>

<https://www.islandbebe.com/expert-advice-malta/mental-health-psychology/maternal-mental-health-help-in-malta/>

<https://deputyprimeminister.gov.mt/en/health-promotion/Documents/library/publications/Perinatal%20Mental%20Health%20Leaflet%20(English)%202018%20FINAL.PDF>

<https://deputyprimeminister.gov.mt/en/Documents/National-Health-Strategies/Mental_Health_Strategy_EN.pdf>

<https://meae.gov.mt/en/Public_Consultations/MEH-HEALTH/Documents/Mental%20Health%20Strategy.pdf>

Monaco

<https://www.legimonaco.mc/305/legismclois.nsf/ViewTNC/E58DE4C92E368740C125773F0037646D!OpenDocument>

<https://en.gouv.mc/Policy-Practice/Social-Affairs-and-Health/News/Multilateral-meeting-to-adopt-Psychological-Stability-and-Wellbeing-mental-health-plan>

<https://en.service-public-particuliers.gouv.mc/Employment/Employees/Leave-and-sickness/Pregnancy-and-maternity-leave>

Montenegro

<https://www.gov.me/cyr/pretraga?page=1&sort=published_at>

<https://www.gov.me/cyr/dokumenta/60af25aa-a65e-4957-84d8-e77a3182ab9b>

<https://wapi.gov.me/download-preview/60af25aa-a65e-4957-84d8-e77a3182ab9b?version=1.0>

<https://www.gov.me/cyr/dokumenta/3c199212-ba43-4dc8-8821-a8d3cfa0b4f4>

<https://www.ugoscgrs.rs/>

[www.mzdravlja.gov.me](http://www.mzdravlja.gov.me)

<https://www.ncbi.nlm.nih.gov/pmc/articles/PMC5619492/>

Netherlands

<https://www.government.nl/topics/mental-health-services/question-and-answer/help-for-mental-health-problems>.

<https://www.government.nl/topics/abortion,>

<https://www.rivm.nl/en/population-screening-programmes>

[https://richtlijnendatabase.nl/richtlijn/antipsychotica_en_niet ssri_antidepressiva_tijdens_zwangerschap_en_lactatie/organisatie_van_zorg_bij_antipsychotica_en_niet-ssri_antidepressiva_tijdens_zwangerschap_en_lactatie/organisatie_van_postpartum_zorg_bij_antipsychotica_en_niet-ssri_antidepressiva_tijdens_zwangerschap_en_lactatie.html](https://richtlijnendatabase.nl/richtlijn/antipsychotica_en_niet%20ssri_antidepressiva_tijdens_zwangerschap_en_lactatie/organisatie_van_zorg_bij_antipsychotica_en_niet-ssri_antidepressiva_tijdens_zwangerschap_en_lactatie/organisatie_van_postpartum_zorg_bij_antipsychotica_en_niet-ssri_antidepressiva_tijdens_zwangerschap_en_lactatie.html)

<https://www.babysensory.nl/postnatal_depression>

<https://www.zorg-en-gezondheid.be/sites/default/files/atoms/files/Richtlijn-perinatale-gezondheid.pdf> -

<https://www.zorg-en-gezondheid.be/sites/default/files/atoms/files/Richtlijn-perinatale-gezondheid.pdf>

<https://www.24baby.nl/zwanger/gezondheid/pop-poli/>

<https://www.cambridge.org/core/journals/bjpsych-international/article/perinatal-mental-health-around-the-world-priorities-for-research-and-service-development-in-the-netherlands/37B91B75398A2F279059EA5A1B1BEA1D>

North Macedonia

<http://zdravstvo.gov.mk/zakoni-2/>

<http://zdravstvo.gov.mk/model-na-statut-na-javni-zdravstveni-ustanovi/>, <http://zdravstvo.gov.mk/wp-content/uploads/2015/08/Postpartalna-psihoza-i-drugi.pdf>

<http://zdravstvo.gov.mk/wp-content/uploads/2020/05/strategija-za-MZ-2018-2025-170718-pf-1.pdf>

Norway

<https://www.legeforeningen.no/foreningsledd/fagmed/norsk-gynekologisk-forening/veiledere/arkiv-utgatte-veiledere/veileder-i-fodselshjelp-2014/30.-depresjoner-i-svangerskapet-og-ammeperioden/>

<https://www.legeforeningen.no/foreningsledd/fagmed/norsk-gynekologisk-forening/veiledere/veileder-i-fodselshjelp/mental-helse-i-svangerskapet/>

[Prop. 1 S (2020–2021) - regjeringen.no](https://www.regjeringen.no/no/dokumenter/prop.-1-s-20202021/id2768429/?ch=1)

[phd-Shakeel-2019.pdf (uio.no)](https://www.duo.uio.no/bitstream/handle/10852/68309/phd-Shakeel-2019.pdf?sequence=1&isAllowed=y)

<https://www.ncbi.nlm.nih.gov/pmc/articles/PMC8554966/>

Portugal

[Saúde mental na gravidez e pós-parto – CH | Tâmega e Sousa (min-saude.pt)](https://www.chts.min-saude.pt/mais-saude/saude-mental/saude-mental-na-gravidez-e-pos-parto/)

Plano Nacional de Saude Mental.pdf

[Projeto em Faro promove saúde mental nas grávidas da pandemia (sapo.pt)](https://barlavento.sapo.pt/destaque/projeto-em-faro-promove-saude-mental-nas-gravidas-da-pandemia)

[Projecto quer promover a saúde mental da mulher na gravidez e pós-parto - Saúde Mental (min-saude.pt)](https://saudemental.min-saude.pt/projecto-quer-promover-a-saude-mental-da-mulher-na-gravidez-e-pos-parto/)

[cn_4_01_dsmia (dgs.pt)](https://www.dgs.pt/directrizes-da-dgs/normas-e-circulares-normativas/circular-normativa-n-4dsmia-de-01032001-pdf.aspx)

[Linha telefónica apoio à saúde mental na gravidez e pós-parto (uptokids.pt)](https://uptokids.pt/linha-telefonica-apoio-a-saude-mental-na-gravidez-e-pos-parto/)

<https://www.portugal.gov.pt/pt/gc22/comunicacao/noticia?i=governo-quer-qualificar-acesso-aos-cuidados-de-saude-mental>

Poland

<https://psychiatria.org.pl/aktualnosci,tekst,74>

<https://docs.google.com/document/d/e/2PACX-1vT9CXAJWNivhzIB_DP4KTPUZZXOqL_1Xb0CrzQUavVoKUseJ9Wqjk577DV52jtHpXFXSMZyACZJuPjz/pub>

<https://www.gov.pl/web/uw-warminsko-mazurski/depresja-poporodowa>

<https://www.gov.pl/web/rpp/narodowy-program-ochrony-zdrowia-psychicznego>

<https://www.ptgin.pl/node/443>

<https://www.gov.pl/attachment/0693bb78-ab71-4edf-b0fa-cc94add596fa>

<https://www.gov.pl/attachment/be370cc4-dc34-4999-9907-0d6d1afed4c>

<https://www.gov.pl/attachment/4a9bd160-e052-4a52-8fd4-b7c546d556f8>

<https://www.pap.pl/mediaroom/1219626%2Ckongres-zdrowia-kobiet-26-28-maja-2022-r.html>

<http://www.ptpol.pl/media/dopobrania/ZG_PTPol_rekomendacje_do_standardow_organizacyjnych_opieki_okoloporodowej.pdf>

<https://rodzicpoludzku.pl/wp-content/uploads/2009/01/problemy_emocjonalne_okres_okoloporodowy-1.pdf>

<https://www.ncbi.nlm.nih.gov/pmc/articles/PMC9517552/#:~:text=The%20situation%20in%20Poland%20changed,is%20a%20huge%20step%20forward>.

Republic of Moldova

<https://msmps.gov.md/legislatie/ghiduri-protocoale-standarde/>

<http://sanatatemintala.md/en/resources>

<https://msmps.gov.md/wp-content/uploads/2020/07/15439-PCN20-27820Tulburari20de20anxietate20la20adult.pdf>

<http://sanatatemintala.md/images/documente/Ghid_urgente_tipar.pdf>

<http://sanatatemintala.md/images/resurse/Deprsia%20rezistenta%20(16.APR.2019).pdf>

<https://www.facebook.com/sanatatemintala.md/>

<https://www.glowm.com/figo-society/id/1135>

<https://msmps.gov.md/wp-content/uploads/2022/01/PA-al-MS-2022-pdf.pdf>

Romania

<https://sgg.gov.ro/1/wp-content/uploads/2016/11/Anexa-Calendar-Strategie.pdf>

<https://mfe.gov.ro/wp-content/uploads/2020/07/5e0bdcbddccca4d66d74ba8c1cee1a68.pdf>

<https://www.ms.ro/wp-content/uploads/2016/10/Anexa-1-Strategia-Nationala-de-Sanatate-2014-2020.pdf>

<https://legislatie.just.ro/Public/DetaliiDocumentAfis/173679>

<https://www.medichub.ro/reviste/ginecologia-ro/depresia-perinatala-id-2884-cmsid-65>

<https://www.medichub.ro/reviste-de-specialitate/medic-ro/depresia-post-partum-preventie-diagnostic-si-abordare-terapeutica-id-4591-cmsid-51>

<https://www.ms.ro/2022/01/13/campania-privind-sanatatea-mintala/>

<https://legislatie.just.ro/Public/DetaliiDocumentAfis/173680>

<https://dspbv.ro/promovarea-sanatatii/sanatatea-mintala-2023/>

<http://old.ms.ro/?pag=136>

Russian Federation

<https://psychiatr.ru/news/1198>

<https://cr.minzdrav.gov.ru/schema/675_1>

<https://psychiatr.ru/news/1198>

<https://cr.minzdrav.gov.ru/schema/301_2>

<https://cr.minzdrav.gov.ru/schema/451_2>

<http://npar.ru/>

<https://roag-portal.ru/clinical_recommendations>

<https://minzdrav.gov.ru/documents?utf8=%E2%9C%93&document_search%5Bq%5D=%D0%B4%D0%B5%D0%BF%D1%80%D0%B5%D1%81%D1%81%D0%B8%D1%8F+&document_search%5Bkind%5D=&document_search%5Bissued_by%5D=&document_search%5Bnumber%5D=&document_search%5Btitle_only%5D=false&document_search%5Bcategory_ids%5D%5B%5D=&document_search%5Bissued_from%5D=&document_search%5Bissued_until%5D=&document_search%5Border%5D=date_desc>

<https://static-0.minzdrav.gov.ru/system/attachments/attaches/000/025/372/original/%D0%9F%D1%80%D0%B8%D0%BA%D0%B0%D0%B7_%D0%9C%D0%B8%D0%BD%D0%B8%D1%81%D1%82%D0%B5%D1%80%D1%81%D1%82%D0%B2%D0%B0_%D0%B7%D0%B4%D1%80%D0%B0%D0%B2%D0%BE%D0%BE%D1%85%D1%80%D0%B0%D0%BD%D0%B5%D0%BD%D0%B8%D1%8F_%D0%A0%D0%A4_%D0%BE%D1%82_20_%D0%B4%D0%B5%D0%BA%D0%B0%D0%B1%D1%80%D1%8F_2012%C2%A0%D0%B3._N%C2%A01219%D0%BD.pdf?1429097626>

<https://static-0.minzdrav.gov.ru/system/attachments/attaches/000/025/377/original/%D0%9F%D1%80%D0%B8%D0%BA%D0%B0%D0%B7_%D0%9C%D0%B8%D0%BD%D0%B8%D1%81%D1%82%D0%B5%D1%80%D1%81%D1%82%D0%B2%D0%B0_%D0%B7%D0%B4%D1%80%D0%B0%D0%B2%D0%BE%D0%BE%D1%85%D1%80%D0%B0%D0%BD%D0%B5%D0%BD%D0%B8%D1%8F_%D0%A0%D0%A4_%D0%BE%D1%82_20_%D0%B4%D0%B5%D0%BA%D0%B0%D0%B1%D1%80%D1%8F_2012%C2%A0%D0%B3._N%C2%A01226%D0%BD.pdf?1429105421>

<https://psychiatr.ru/download/4070?view=1&name=%D0%A1%D0%A2%D0%A0%D0%90%D0%A2%D0%95%D0%93%D0%98%D0%AF_04.19_%D0%BF%D1%80%D0%BE%D0%B5%D0%BA%D1%82.pdf>

San Marino

17126967PianoSanitario20.pdf

<https://www.sanita.sm/pub1/SanitaSM/Segreteria-di-Stato/Staff-Segreteria-Sanita.html>

<https://www.iss.sm/on-line/home/dedicato-a/articolo49000314.html#mentale>

<https://www.domusmedica.sm/attivita/psicologia/>

Serbia

<http://demo.paragraf.rs/demo/combined/Old/t/t2019_12/t12_0013.htm>

<https://www.zdravlje.gov.rs/tekst/335903/nacionalni-vodici-dobre-klinicke-prakse.php>

<https://www.zdravlje.gov.rs/view_file.php?file_id=702&cache=sr>

<http://www.imh.org.rs/data/flajer.pdf>

<http://ups-spa.org/>

<https://www.ugoscgrs.rs/>

<https://www.researchgate.net/publication/262842355_Perinatalna_psihijatrija_-_smernice_u_klinickoj_praksi>

<https://www.vladars.net/sr-SP-Cyrl/Vlada/Ministarstva/MZSZ/Documents/mentalno%20zdravlje%20ENG.pdf>

<http://imh.org.rs/page.php?id=23>

Slovakia

<http://www.psychiatry.sk/medzinarodne-dokumenty/zelena-kniha>

<http://www.psychiatry.sk/cms/File/reforma/k%20reforme%20psychiatrickej%20starostlivosti_final.pdf>

<http://sgps.sk/>

<https://lekar.sk/clanok/poporodna-depresia>

<http://www.psychiatry.sk/>

Slovenia

<http://www.zpsih.si/strokovno/smernice>

<http://www.zpsih.si/strokovno/media/documents/Smernice_depresija2.pdf>

[http://www.pisrs.si/Pis.web/pregledPredpisa?id=ZAKO2157#](http://www.pisrs.si/Pis.web/pregledPredpisa?id=ZAKO2157)

<https://www.gov.si/teme/dusevno-zdravje/>

<https://www.gov.si/assets/ministrstva/MZ/DOKUMENTI/Preventiva-in-skrb-za-zdravje/Varovanje-in-krepitev-zdravja/dusevno-zdravje/KamInKakoPom2012.pdf>

<https://www.gov.si/assets/ministrstva/MZ/DOKUMENTI/staro/Preventiva-in-skrb-za-zdravje/Varovanje-in-krepitev-zdravja/dusevno-zdravje/dusevno-zdravje/Akcijski-nacrt-za-dusevno-zdravje-2022-2023-310322.pdf>

<https://www.szd.si/sekcije-in-zdruzenja/psihiatrija/zdruzenje-psihiatrov/>

<http://www.pisrs.si/Pis.web/pregledPredpisa?id=RESO120>

<https://www.zadusevnozdravje.si/wp-content/uploads/2021/03/Mira-resolucija-SLO_splet-2021.pdf>

https://promentum.si/wp-content/uploads/2021/11/Dusevno_zdravje_v_obporodnem_obdobju_prirocnik.pdf

<https://nijz.si/wp-content/uploads/2022/11/dusevno_zdravje_in_nosecnost_porod_ter_zgodnje_starsevstvo.pdf>

Spain

[Maqueta_OMS.indd (sanidad.gob.es)](https://www.sanidad.gob.es/organizacion/sns/planCalidadSNS/pdf/excelencia/salud_mental/opsc_est13.pdf.pdf)

[Actualizada la guía del NICE sobre atención de la salud mental en el embarazo y posparto (infocop.es)](https://www.infocop.es/view_article.asp?id=7457)

[12_junio_plan_de_salud_mental_2018-2020.pdf (comunidad.madrid)](https://www.comunidad.madrid/sites/default/files/doc/sanidad/asis/12_junio_plan_de_salud_mental_2018-2020.pdf)

<https://www.sanidad.gob.es/organizacion/sns/planCalidadSNS/pdf/equidad/ENSSR_English.pdf>

<https://www.clinicbarcelona.org/en/assistance/diseases/postnatal-depression/treatment>

<https://www.clinicbarcelona.org/en/assistance/diseases/postnatal-depression/diagnosis>

<https://www4.hcdn.gob.ar/dependencias/dsecretaria/Periodo2021/PDF2021/TP2021/0406-D-2021.pdf>

<https://www.elsevier.es/es-revista-revista-psiquiatria-salud-mental--286-avance-resumen-new-units-for-perinatal-mental-S1888989122000751>

Sweden

<https://lakartidningen.se/wp-content/uploads/EditorialFiles/ES/%5BE9ES%5D/E9ES.pdf>

<https://vardgivare.skane.se/siteassets/1.-vardriktlinjer/regionala-riktlinjer---fillistning/psykisk-ohalsa-i-samband-med-graviditet-riktlinje.pd>

<https://lakartidningen.se/klinik-och-vetenskap-1/artiklar-1/temaartikel/2018/09/battre-perinatalpsykiatrisk-vard-genom-integrerat-samarbete>

<https://www.socialstyrelsen.se/en/about-us/healthcare-for-visitors-to-sweden/about-the-swedish-healthcare-system/>

<https://www.sfog.se/start/intressegrupper/obstetriker-och-gynekologer-under-utbildning/ogu-dagarna-samtliga/oerebro-2019/>

Switzerland

Rapport_Sante_psychique_Suisse (1).pdf

[Rapport_GT_v6 (ge.ch)](https://www.ge.ch/document/7565/telecharger)

[www.hug-ge.ch/perinatal-depression/prenatal-interview](http://www.hug-ge.ch/perinatal-depression/prenatal-interview)

[Brochure_PSCH_2021_08_-_Soutenir_la_sante_psychique_des_parents.pdf (promotionsante.ch)](https://promotionsante.ch/assets/public/documents/fr/5-grundlagen/publikationen/psychische-gesundheit/empfehlungen/kinder-und-jugendliche/Brochure_PSCH_2021_08_-_Soutenir_la_sante_psychique_des_parents.pdf)

[SantéPsy.ch - Resources available to future parents and parents of young people (santepsy.ch)](https://www.santepsy.ch/fr/pages/ressources-a-disposition-des-futurs-parents-et-parents-de-jeunes-enfants-1011)

<https://www.bag.admin.ch/bag/en/home/das-bag/aktuell/news/news-03-12-2020.html>

<https://www.sggg.ch/fileadmin/user_upload/Dokumente/3_Fachinformationen/4_Patienteninformationsblaetter/D_Informationsflyer_Postpartale_Depression.pdf>

<https://www.promentesana.ch/>

<https://postpartale-depression.ch/de/ueber-uns/ziele-und-taetigkeiten.html>

<https://www.swissinfo.ch/eng/how-an-app-is-helping-women-self-diagnose-perinatal-depression/46515038>

https://gesundheitsfoerderung.ch/sites/default/files/migration/documents/Broschuere_GFCH_2021_08_-_Die_psychische_Gesundheit_von_Eltern_unterstuetzen.pdf

Tajikistan

<http://www.moh.tj/>

<https://adbmch.tj/>

<https://niiagip.tj/>

<https://www.undp.org/sites/g/files/zskgke326/files/migration/tj/UNDP-TJK-Pub_2021_06-1_Final_SurveyTJ_12102020.pdf>

Turkey

<https://nazanaydin.com.tr/perinatal-psychiatric-approach>

<https://tpdyayin.psikiyatri.org.tr/Book.aspx?book=128> <https://www.saglik.gov.tr/TR,11186/yonergeler.html>

<https://psikiyatri.org.tr/tpd-kutuphanesi/20>

<https://www.tjod.org/category/dosyalarimiz/yonergeler/>

<https://www.anneruhsagligi.com/upload/content/files/TUSEB-TACESE_Sunum_Kitapcigi.pdf>

<https://stratejikplan.saglik.gov.tr/files/TC-Saglik-Bakanligi-2019-2023-Stratejik-Plan-Web-Katalog.pdf>

<https://hsgm.saglik.gov.tr/depo/birimler/Ruh_Sagligi_Db/yayinlarimiz/URSEP_Baski.pdf> -

Turkmenistan

<https://www.saglykhm.gov.tm/home>

<https://www.mfa.gov.tm/en/articles/7>

Ukraine

<https://moz.gov.ua/article/news/ofis-pershoi-ledi-ukraini-zapuskae-zagalnonacionalnu-iniciativu-schodo-zmicnennja-psihichnogo-zdorov%e2%80%99ja-ta-psihosocialnoi-pidtrimki-gromadjan>

<https://moz.gov.ua/article/news/rozpochalas-gromadska-diskusija-schodo-programi-ohoroni-psihichnogo-zdorov%e2%80%99ja-v-ukraini->

<https://www.mh4u.in.ua/wp-content/uploads/2021/09/opz-v-gromadah-posibnyk-klymchuk-suvalo.pdf>

<http://www.aagu.com.ua/en/methodological.php>

<https://inpn.org.ua/organization-and-methodology-activities>

<https://www.mh4u.in.ua/wp-content/uploads/2021/09/opz-v-gromadah-posibnyk-klymchuk-suvalo.pdf>

<https://www.dec.gov.ua/wp-content/uploads/2019/11/akn_opz.pdf>

United Kingdom

[Recommendations | Antenatal and postnatal mental health: clinical management and service guidance | Guidance | NICE](https://www.nice.org.uk/guidance/cg192/chapter/Recommendations)

[Mental health problems and pregnancy - NHS (www.nhs.uk)](https://www.nhs.uk/pregnancy/keeping-well/mental-health/)

215869_418e08a9-1ae2-4b06-9c15-a2063ac05f05.pdf

[Consensus-Statement-Perinatal-Mental-Health-northern-ireland-april-2019-MMHA.pdf (maternalmentalhealthalliance.org)](https://maternalmentalhealthalliance.org/wp-content/uploads/Consensus-Statement-Perinatal-Mental-Health-northern-ireland-april-2019-MMHA.pdf)

<https://www.nhs.uk/mental-health/conditions/post-natal-depression/treatment/>

<https://www.app-network.org/about-us/>

<https://pandasfoundation.org.uk/>

<https://www.nct.org.uk/about-us/vision-mission-and-goal>

<https://apni.org/our-function/>

Uzbekistan

<https://psychiatry.medped.tma.uz/uz/uslubij-ishlar>

<https://roddom6.uz/>

<http://minzdrav.uz/>

<https://nrm.uz/contentf?doc=579732_koncepciya_razvitiya_slujby_ohrany_psihicheskogo_zdorovya_naseleniya_respubliki_uzbekistan_na_2019-2025_gody_(prilojenie_n_1_k_postanovleniyu_prezidenta_ruz_ot_13_02_2019_g_n_pp-4190)&products=1_vse_zakonodatelstvo_uzbekistana>

<https://tma.uz/2019/03/01/ob-utverzhdenii-kontseptsii-razvitiya-sluzhby-ohrany-psihicheskogo-zdorovya-naseleniya-respubliki-uzbekistan-na-2019-2025-gody/?lang=ru> –

<https://lex.uz/ru/docs/-6505600>

<https://www.ncbi.nlm.nih.gov/pmc/articles/PMC9295855/>

International

<https://www.euro.who.int/__data/assets/pdf_file/0004/333913/strategy-womens-health-ru.pdf>

<https://www.unfpa.org/data/transparency-portal/unfpa-belarus>

<https://www.ilo.org/wcmsp5/groups/public/---ed_norm/---normes/documents/publication/wcms_553511.pdf>

<https://ec.europa.eu/info/sites/default/files/mntfs2030.pdf>

<https://cdn.who.int/media/docs/default-source/mental-health/who-aims-country-reports/kyrgyzstan_who_aims_report.pdf?sfvrsn=d89fa0c4_3&download=true>

<https://www.figo.org/kyrgyz-association-obstetricians-gynecologists-and-neonatologists-koagn>

<https://www.rbfhealth.org/sites/rbf/files/Knowledge-Brief-Mama-Natalie-Neo-Natalie.pdf>

<https://www.euro.who.int/en/countries/albania/news/news/2020/11/regional-directors-visit-cements-stronger-cooperation-on-health-in-albania>
